# Surgical Management of Anterior Cruciate Ligament Rupture in Skeletally Immature Adolescents: A Bilateral Transphyseal All‐Inside Technique With Patient‐Specific Bone Tunnels and Semitendinosus Autograft

**DOI:** 10.1002/atn2.70051

**Published:** 2026-05-12

**Authors:** Zeming Li, Junling Luo, Chuanliang Chen, Shifeng Zhong, Hao Feng, Hongtao Wang, Jun Yao

**Affiliations:** ^1^ Department of Bone & Joint Surgery The First Affiliated Hospital of Guangxi Medical University Guangxi Medical University Nanning China

## Abstract

In recent years, with the widespread participation in athletic activities, the incidence of anterior cruciate ligament (ACL) ruptures among adolescent populations has shown an increasing trend. Without timely intervention, this condition can lead to meniscal tears, accelerated articular cartilage wear, and early‐onset osteoarthritis, severely compromising knee joint development and function. Due to the presence of open physis, standard ACL reconstruction techniques used in adults may jeopardize the physeal structure during tunnel preparation and graft placement, thereby interfering with normal lower limb growth and potentially resulting in limb length discrepancies or angular deformities. Therefore, this study adopts a personalized and precision treatment strategy, using preoperative magnetic resonance imaging (MRI) for systematic quantitative analysis of each patient's knee joint, combined with an all‐inside transphyseal reconstruction technique. This approach aims to effectively restore ACL function while minimizing the risk of physeal injury. This image‐based, patient‐specific bone tunnel planning strategy is designed to protect the physeal integrity while addressing the biomechanical stability requirements of the knee, offering a safe and effective treatment option for adolescent patients.

## Graphical abstract

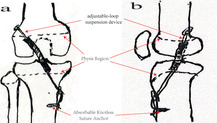

**VIDEO 1:** Surgical demonstration: anatomical anterior cruciate ligament (ACL) reconstruction technique in a right knee. This procedure shows the step‐by‐step technique for anatomical ACL reconstruction. The patient is placed in the supine position. The arthroscope is introduced through the anterolateral portal for visualization, and the anteromedial portal is used as the working portal. **Step 1:** Creation of the femoral tunnel (knee flexed at 120°). Based on the preoperatively measured distance from the center of the femoral ACL footprint to the posterior wall of the lateral femoral condyle (axial magnetic resonance imaging (MRI)), a 6 mm femoral aimer is selected. The femoral aimer is introduced through the anteromedial portal and positioned to form an approximately 45° angle relative to the medial aspect of the lateral femoral condyle (coronal MRI measurement: the angle between the medial aspect of the lateral femoral condyle and a line parallel to the ACL fibers). A 2.0 mm Kirschner‐wire followed by a 4.5 mm EndoButton reamer is advanced through the aimer in a posteroinferior‐to‐anterosuperior direction to create a minimally invasive transphyseal tunnel for the femoral‐sided adjustable suspensory fixation device. Subsequently, a 7.0 mm reamer is used over the Kirschner‐wire to create the final femoral tunnel, approximately 17 mm in length (coronal MRI measurement: distance from the femoral ACL footprint center to the physeal line along the ~45° angle), to accommodate the femoral end of the tendon graft. **Step 2:** Creation of the tibial tunnel (knee flexed at 90°). A tibial aimer is introduced through the anteromedial portal and angled approximately 75° relative to the tibial plateau (sagittal MRI measurement) and approximately 25° relative to the long axis of the tibia (coronal MRI measurement). A 3.5 mm retrograde drill is used through the aimer to create a minimally invasive transphyseal tunnel in a posteroinferior‐to‐anterosuperior direction for the tibial‐sided adjustable suspensory fixation device. Subsequently, a 7.0 mm drill head is deployed from the tip of the retrograde drill to create the final tibial tunnel, approximately 18 mm in length (coronal/sagittal MRI measurement: distance from the tibial ACL footprint center to the physeal line along the established angles), in a reverse direction to accommodate the tibial end of the tendon graft. **Step 3:** Graft passage and fixation. A passing wire with an eyelet is used to shuttle two No. 5 ETHIBOND sutures through the femoral and tibial tunnels, respectively, to serve as relay sutures. The adjustable suspensory fixation device is first pulled into the femoral tunnel using the relay sutures, and the EndoButton is flipped and seated against the lateral femoral cortex. The graft is then advanced into the joint by repeatedly tensioning the device's adjustable sutures until the marked line on the graft reaches the femoral tunnel aperture. Using the same method, the other end of the graft is pulled into the tibial tunnel. Finally, a knotless anchor is used on the tibial side to secure the tail ends of the adjustable sutures approximately 20 mm from the tibial suspensory button, achieving a hybrid fixation construct. This completes the ACL reconstruction. Video content can be viewed at https://doi.org/10.1002/atn2.70051.

With the widespread popularity of sports activities and increased athletic intensity, the incidence of anterior cruciate ligament (ACL) ruptures among adolescents has been progressively rising.^1^, ^2^, ^3^, ^4^ Since patients in this age group are in a critical stage of skeletal growth and development—with open physis.^5^ Current literature primarily describes three surgical techniques to minimize physeal involvement: transphyseal (standard complete transphyseal, partial transphyseal), all‐epiphyseal, and physeal‐sparing techniques.^1^, ^3^, ^6^ Although these methods aim to avoid physeal damage, they do not tailor the bone tunnel design to individual patient anatomy, which may increase the risk of physeal injury. Therefore, to minimize physeal disruption while providing robust ACL graft fixation, the study describes a treatment principle of “individualized bone tunnel planning, maximal bone preservation, and minimal physeal intervention” for skeletally immature adolescents, along with an individualized transphyseal all‐inside ACL reconstruction. This technique uses preoperative magingmagnetic resonance imaging (MRI) to measure the ACL inclination angle and the distance from the ACL footprint to the physeal line, enabling patient‐specific tunnel trajectory planning. The all‐inside transphyseal technique (Figure 1) is then employed for ACL reconstruction, preserving greater bone stock and reducing intervention in the epiphyseal region, thereby offering a surgical option for ACL reconstruction in patients with open physis (Video 1).

**FIGURE 1:**
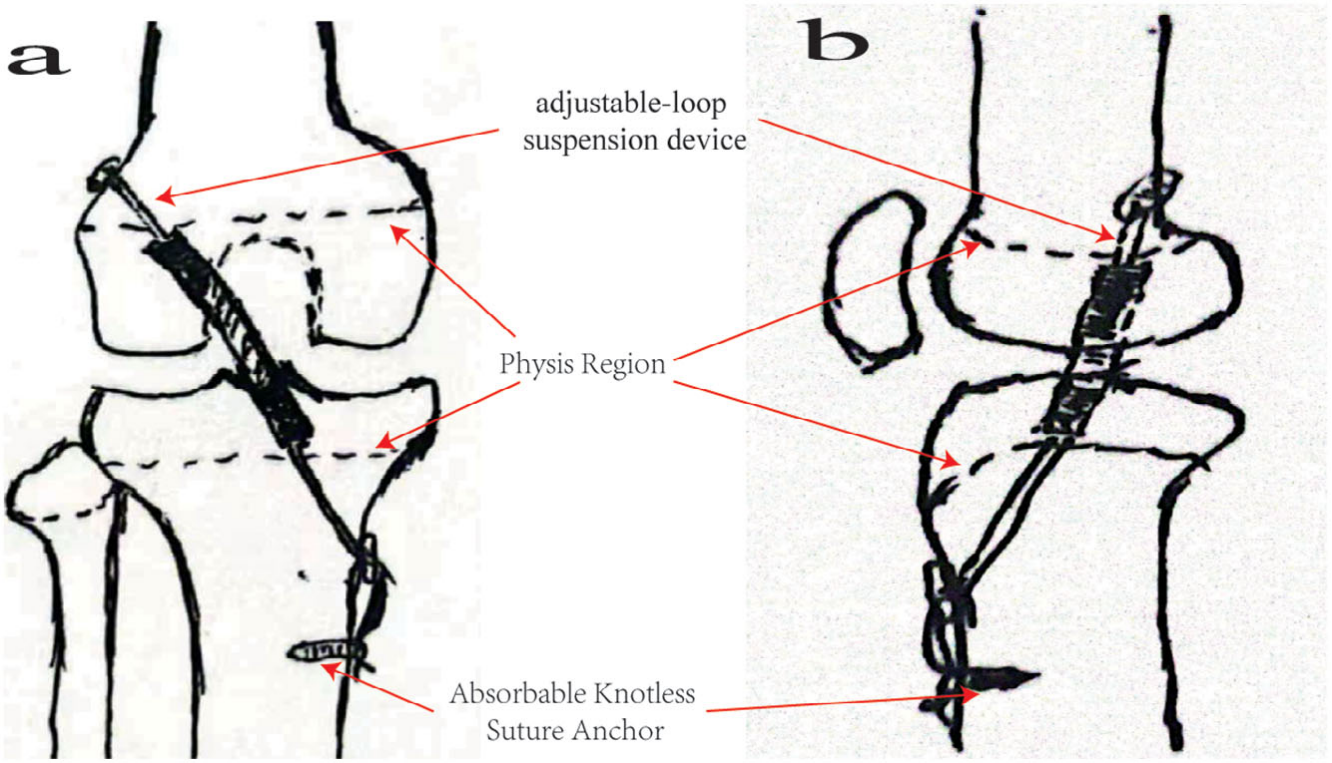
Schematic of the bilateral transphyseal all‐inside technique with patient‐specific bone tunnel planning. (a) represents the frontal view. (b) represents the side view.

## SURGICAL TECHNIQUE

**FIGURE 2:**
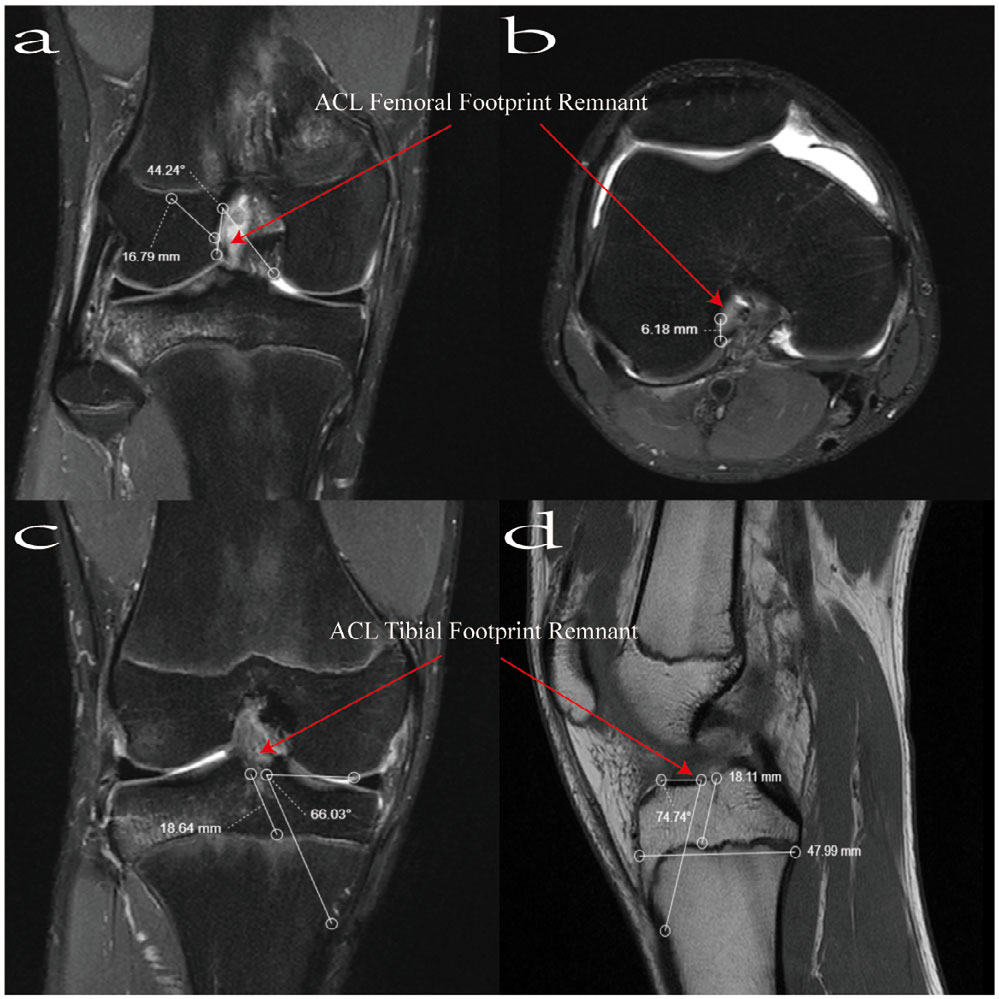
Using magnetic resonance imaging (MRI) of a right knee with an anterior cruciate ligament (ACL) rupture as an example: (a,b) are used to guide the creation of the femoral bone tunnel. (a) Measurement on coronal T1‐weighted MRI: The angle formed between the medial aspect of the lateral femoral condyle and a line parallel to the ACL fiber bundle (approximately 44.24°) is measured. This angle is used to guide the direction for creating both the minimally invasive tunnel and the graft tunnel on the femoral side. Additionally, the distance from the center of the femoral ACL footprint to the physeal line (16.79 mm) is measured. This distance determines the length of the graft tunnel on the femoral side, ensuring it stops before reaching the physis. (b) Measurement on axial T1‐weighted MRI: The distance from the center of the femoral ACL footprint to the posterior wall of the lateral femoral condyle (6.18 mm) is measured. This distance guides the selection of the appropriately sized femoral aimer. (c,d) are used to guide the creation of the tibial bone tunnel. (c) Measurement on coronal T1‐weighted MRI: The angle between the long axis of the tibia and a line parallel to the ACL fiber bundle (90°‐66.03° = 23.97°) is measured. This angle guides the placement angle of the tibial aimer in the sagittal plane. Furthermore, the distance from the center of the tibial ACL footprint to the physeal line (18.64 mm) is measured in the coronal plane. This distance dictates the length of the graft tunnel on the tibial side, ensuring it stops before reaching the physis. (d) Measurement on sagittal T2‐weighted MRI: The angle between the tibial plateau horizontal line and a line parallel to the ACL fiber bundle (74.74°) is measured. This angle guides the placement angle of the tibial aimer in the horizontal (axial) plane. Additionally, the distance from the center of the tibial ACL footprint to the physeal line (18.11 mm) is measured in the sagittal plane. This distance is used to define the length of the graft tunnel on the tibial side, ensuring it stops before reaching the physis.

Preoperative MRI measurements in the coronal, sagittal, and axial planes are performed to assess the ACL inclination angle as well as the distances from the femoral and tibial ACL footprint centers to the physeal line (Figure 2), in preparation for creating patient‐specific bone tunnels that would avoid damaging more physis structures.

**FIGURE 3:**
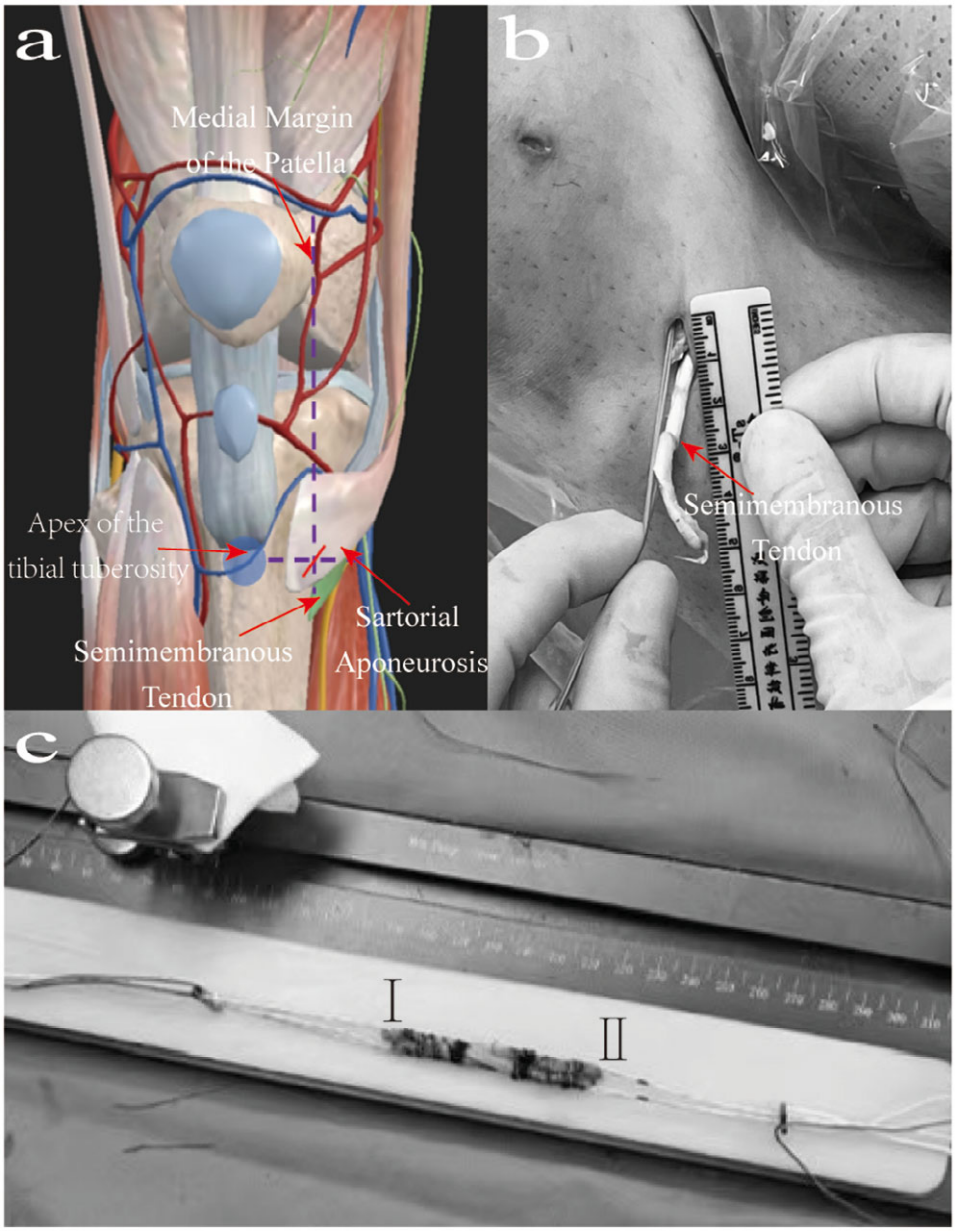
(a) The planned incision for harvesting the semitendinosus tendon: A 1.5 cm oblique incision is made, centered over the intersection point of the longitudinal extension line of the medial border of the patella and the horizontal line passing through the most prominent point of the tibial tuberosity. (b) The harvested semitendinosus tendon. (c) The prepared semitendinosus autograft for anterior cruciate ligament (ACL) reconstruction. The graft has a total length of 60 mm and a diameter of 7.0 mm. End I is the femoral‐side tendon bundle, approximately 17 mm long, and End II is the tibial‐side tendon bundle, 18 mm long.

**FIGURE 4:**
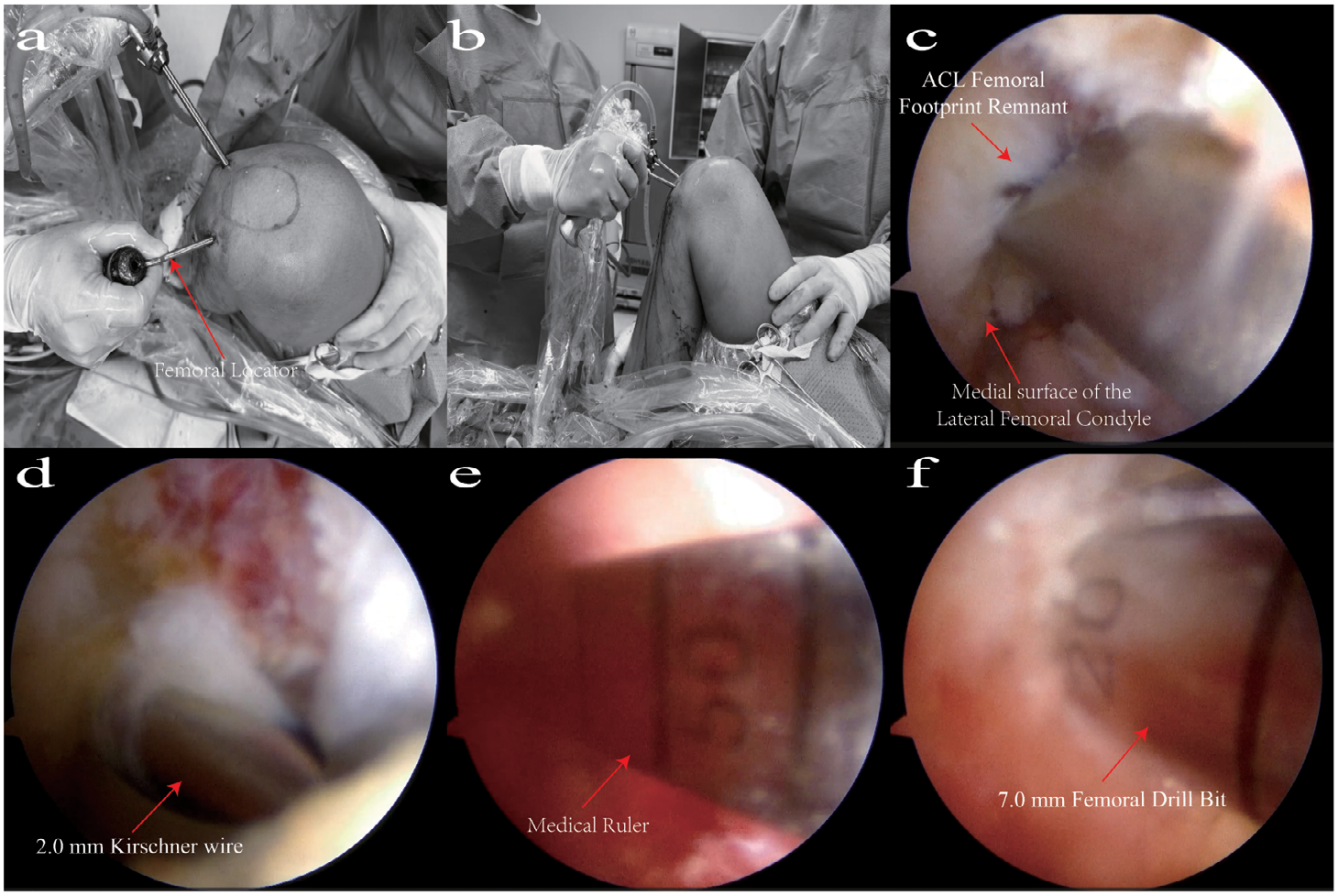
Surgical demonstration: anterior cruciate ligament (ACL) reconstruction in a right knee. This procedure shows the creation of a patient‐specific femoral bone tunnel based on preoperative magnetic resonance imaging (MRI) measurements. Patient position: supine. (a,b) External macroscopic views of the setup. The right knee is flexed to 120°. The view is from the anterolateral portal, with the anteromedial portal serving as the working portal. (c,f) Illustrate the intra‐articular steps under arthroscopy. (c) A femoral aimer (offset: 6 mm) is introduced through the anteromedial portal to guide the drilling direction along the planned 45° angle. (d) A 2.0 mm Kirschner wire followed by a 4.5 mm EndoButton reamer is advanced through the positioned aimer to create a minimally invasive transphyseal tunnel. (e) A depth gauge is used to measure the full length of the transphyseal tunnel. (f) A 2.0 mm Kirschner wirewire is first placed into the created transphyseal tunnel to serve as a guide. Then, a larger 7.0 mm diameter reamer is used to create the final bone tunnel, which accommodates the tendon graft and promotes femoral‐sided tendon‐to‐bone healing.

**FIGURE 5:**
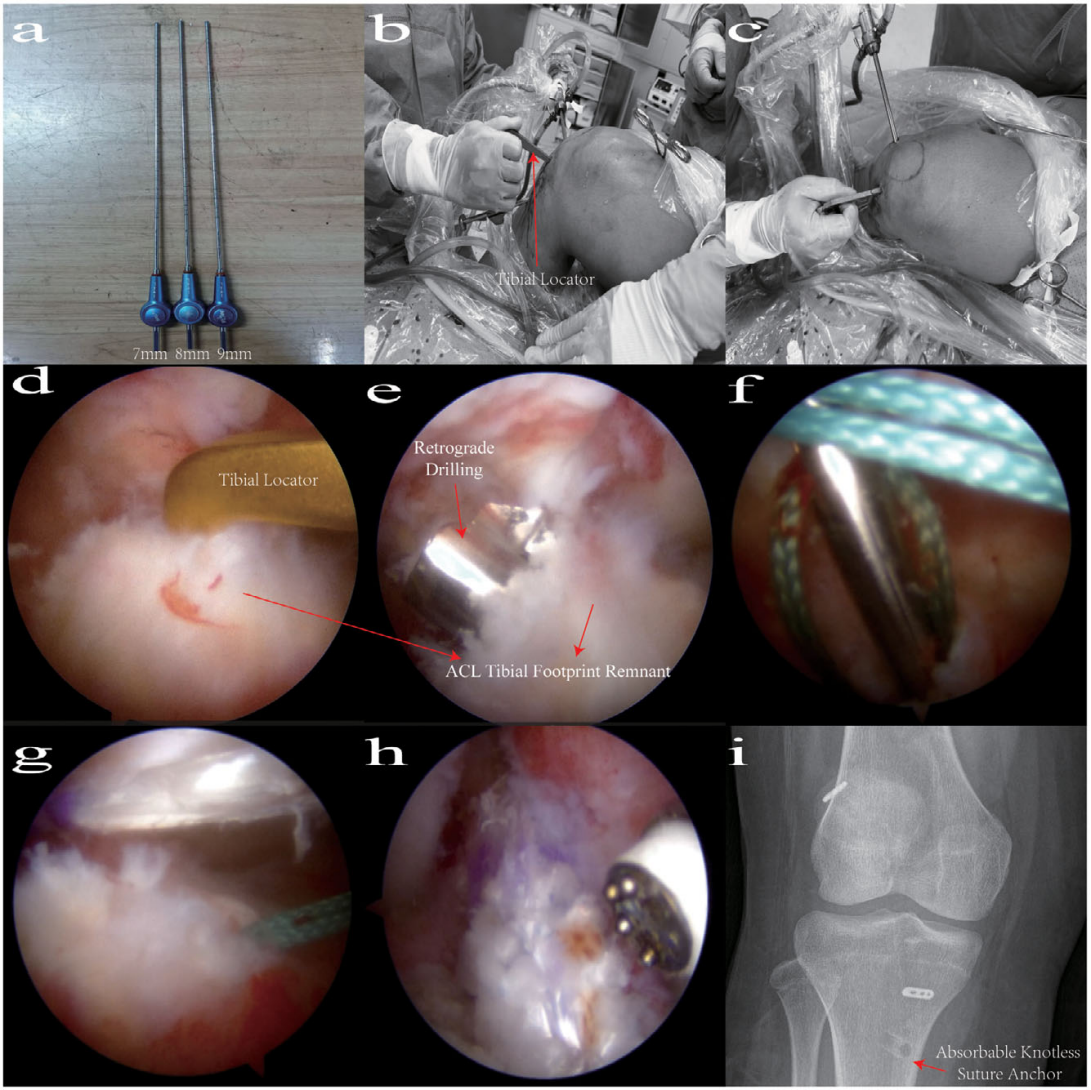
Surgical demonstration: anterior cruciate ligament (ACL) reconstruction in a right knee—tibial tunnel preparation. This procedure shows the creation of a patient‐specific tibial bone tunnel based on preoperative magnetic resonance imaging (MRI) measurements. Patient position: supine. (a) shows the dimensions of the retrograde drill used for the tibial tunnel. (b,c) are external macroscopic views of the setup. The right knee is flexed to 90 degrees. The view is from the anterolateral portal, with the anteromedial portal serving as the working portal. (d‐h) illustrate the intra‐articular steps under arthroscopy. (d) A tibial aimer is introduced through the anteromedial portal, and its angle is adjusted: it forms approximately a 75° angle relative to the tibial plateau in the sagittal plane and approximately a 25° angle relative to the long axis of the tibia in the coronal plane. These combined angles guide the creation of the minimally invasive tibial tunnel. (e) A 3.5 mm retrograde drill is first used through the positioned aimer to create a minimally invasive transphyseal tunnel. Subsequently, a 7.0 mm drill head is deployed from the tip of this retrograde drill to create a larger tunnel (approximately 18 mm in length) in the reverse direction along the same trajectory. This larger tunnel accommodates the tendon graft and promotes tibial‐sided tendon‐to‐bone healing. (f,g) A passing wire with an eyelet tip is used to pull two No. 5 ETHIBOND sutures into the femoral and tibial tunnels, respectively, serving as shuttle sutures. These are used to pull the adjustable suspensory fixation device and the tendon graft into the bone tunnels. (h) After tensioning the adjustable suspensory fixation devices on both ends, the intra‐articular portion of the tendon graft is viewed arthroscopically. (i) Postoperative X‐ray.

Under general anesthesia, the patient is positioned supine and the surgical field is prepared and draped in a standard sterile fashion. Standard anteromedial and anterolateral arthroscopic portals are established. The arthroscope is introduced to examine the ACL for rupture and to evaluate for any concomitant meniscal or chondral injuries. Firstly, a serrated shaver blade (diameter 4.2 mm, work length 132 mm, AccuBlade, Rejoin, Hangzhou, Zhejiang, China) is used to debride hypertrophic synovial tissue and perform a synovectomy in the patellofemoral joint space and the anterior compartment of the knee. A radiofrequency ablation probe (model BDS313, BONSS, Taizhou, Jiangsu, China) is then employed to coagulate the synovial tissue and achieve hemostasis. Subsequently, a meniscal repair system (Fast‐Fix 360, Smith & Nephew, Watford, Hertfordshire, United Kingdom) is used to suture the torn meniscus. Third, a 1.5 cm oblique incision is made at the intersection of the horizontal line at the most prominent point of the tibial tuberosity and the vertical line along the medial border of the patella (Figure 3a). The skin and fascia are incised, exposing the obliquely oriented sartorial fascia. Upon division of this fascial layer, the underlying semitendinosus tendon is identified. The intertendinous membranous tissue and fascial attachments are carefully released. A tendon harvester is introduced and advanced proximally toward the musculotendinous junction to harvest the semitendinosus tendon along with its periosteal extension (Figure 3b). The graft is prepared to a length of 60 to 65 mm and a diameter of 7.0 mm. Both ends of the tendon graft are whip‐stitched with #5 ETHIBOND suture (Ethicon, Somerville, NJ) for 15 to 20 mm in length each (Figure 3c). Femoral tunnel creation: The knee is flexed to 120°. A 6‐mm femoral aimer is introduced and positioned at approximately 45° to the medial aspect of the lateral femoral condyle. The midpoint of the femoral tunnel is located at the ACL remnant on the medial side of the femoral condyle, approximately 6 mm from the posterior wall of the lateral femoral condyle (Figure 4a‐c). A 2.0‐mm Kirschner‐wire is first drilled from inferomedial to superolateral. The Kirschner‐wire is then over‐drilled using a 4.5‐mm EndoButton reamer (Smith & Nephew, Watford, Hertfordshire, United Kingdom) to penetrate the lateral femoral cortex, which results in the formation of a minimally invasive bone tunnel that penetrates through the femur physis. The total length of the femoral tunnel is measured to be 42 mm using a depth gauge. A 7.0‐mm cannulated reamer is used to create a femoral tunnel approximately 17 mm in length (Figure 4d‐f). Tibial tunnel preparation: A retrograde drill (Retrograde Drilling Type I, Wu Yang, Hefei, Anhui, China) is used for tibial tunnel preparation (Figure 5a). With the knee flexed at 90°, an ACL tibial guide is inserted into the joint and set at approximately 25° to the long axis of the tibia and 75° relative to the tibial plateau. The tunnel is drilled as perpendicularly as possible to the physeal line. The center of the tibial tunnel is positioned at the native ACL tibial remnant (Figure 5b‐d). A 3.5‐mm retrograde drill bit is advanced from inferomedial to superolateral into the joint cavity, which results in the formation of a minimally invasive bone tunnel that penetrates through the tibia physis. The drill is then expanded to 7.0 mm, and a retrograde tibial tunnel approximately 18 mm in length is created (Figure 5e). Graft passage and fixation (Figure 5f‐h): The adjustable‐loop suspensory fixation device is first passed through the femoral minimally invasive bone tunnel via the anteromedial portal using a passing suture. The button is flipped and engaged on the cortical surface at the external aperture of the tunnel. The graft is then tensioned and pulled into the femoral tunnel. The same procedure is repeated to pass the free end of the graft into the tibial minimally invasive bone tunnel. Care must be taken to advance the tendon graft precisely to the marking line, avoiding any traversal of the physis. The graft is tensioned, and the knee is cycled through full range of motion to assess for impingement or graft capture. While maintaining tension, a knotless suture anchor (BioComposite SwiveLock C, Closed Eyelet, 4.75 × 19.1 mm, Arthrex, Naples, FL) is used to fix the sutures approximately 20 mm from the adjustable button, providing additional fixation. Final fluoroscopy confirmed the position of adjustable‐loop suspension device (Figure 5i). ACL reconstruction is thus completed.

**FIGURE 6:**
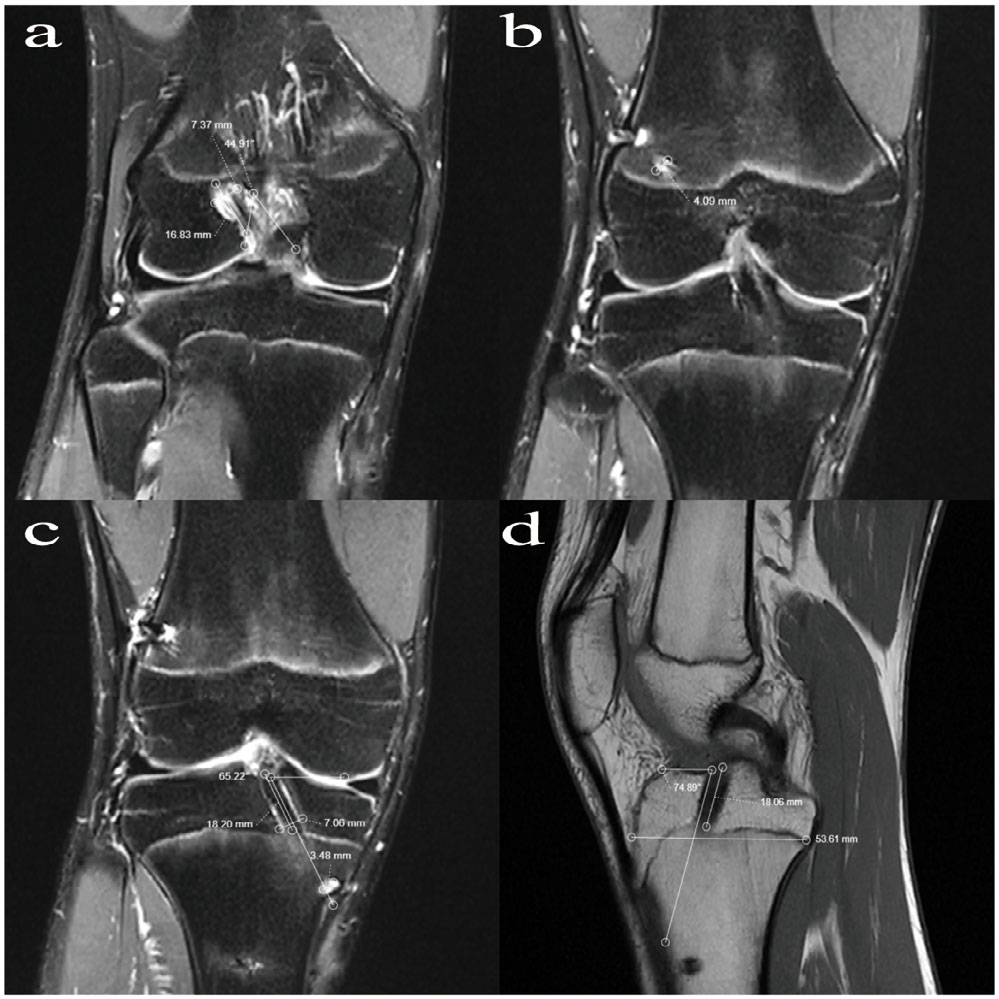
Right Knee. Postoperative magnetic resonance imaging at 3‐month follow‐up showing consistency between the actual bone tunnel trajectory and the preoperatively planned individualized tunnel pathway.

Postoperative evaluation: A 3‐month postoperative MRI is performed to assess graft maturation and the position of the individualized bone tunnels (Figure 6). Follow‐up imaging showed well‐healed minimally invasive bone tunnels across the femoral and tibial physis, with absence of graft traversal and no space‐occupying effect within the physis region.

## DISCUSSION

For skeletally immature adolescents, failure to promptly address an ACL rupture can lead to recurrent joint instability, secondary meniscal tears, or early‐onset osteoarthritis, which significantly impairs athletic participation and quality of life.^7^ Therefore, early surgical intervention is crucial for adolescent ACL injuries. However, the unique anatomy of open physis in this population poses distinct challenges for ACL reconstruction techniques.

Currently, reported techniques for adolescent ACL reconstruction primarily fall into three categories: transphyseal techniques (including standard complete transphyseal and partial transphyseal techniques), all‐epiphyseal techniques, and physeal‐sparing techniques.^1^, ^3^, ^5^, ^6^ Each of these techniques has its own advantages and limitations, and all lack a patient‐specific bone tunnel trajectory design. The standard complete transphyseal technique allows for anatomical reconstruction, provides excellent biomechanical performance, and is a well‐established procedure similar to that used in adults. However, this technique requires the creation of tunnels that traverse the physis, resulting in the largest cross‐physeal area. Furthermore, the graft and the interference screws used for fixation often cross the physeal region.^8^ Consequently, it carries a significant risk of extensive physeal intervention and damage. The partial transphyseal technique involves creating a transphyseal tunnel on the tibial side, while on the femoral side, a physeal‐sparing approach is adopted—such as fixation at the lateral femoral condyle apex or within the epiphysis.^6^, ^8^, ^9^ This approach minimizes the risk of physeal injury on the femoral side; however, the graft and interference screw within the tibial tunnel still substantially interfere with the physis. Additionally, femoral‐sided apex fixation does not achieve true anatomical reconstruction, while epiphyseal fixation—due to its proximity to the physis—carries a risk of extensive transverse physeal damage. Other challenges include high technical difficulty, a steep learning curve, limited bone stock, and nonanatomical graft placement. The all‐epiphyseal technique offers the key advantage that both tibial and femoral tunnels remain entirely within the epiphysis without penetrating the physis,^4^, ^6^, ^9^ thereby enabling anatomical reconstruction. Theoretically, this eliminates the risk of limb length discrepancy or angular deformity resulting from surgical intervention. Nevertheless, the procedure is confined to the underdeveloped epiphysis near the physis, demanding extremely high precision in tunnel positioning and trajectory. This results in considerable technical complexity, a prolonged learning process, and elevated risks of iatrogenic physeal damage and tunnel breach. Moreover, limited bone stock within the epiphysis may preclude the use of sufficiently large grafts, and restrictions in graft length and fixation methods increase the risk of graft failure.^6^, ^8^, ^10^, ^11^ The physeal‐sparing technique is exemplified by the modified MacIntosh procedure, an extra‐articular reconstruction method where the graft is fixed externally to the femur and tibia.^12^ Since all steps are performed outside the bone, this technique entirely avoids the risk of drill‐induced physeal injury and completely eliminates the potential for growth arrest. However, it is associated with several drawbacks, including nonanatomical reconstruction, postoperative joint stiffness, inferior fixation strength, and a higher risk of graft failure.^13^

To achieve an optimal balance among anatomical ACL reconstruction, physeal preservation, and complication control, this study developed an individualized bone tunnel transphyseal all‐inside ACL reconstruction technique featuring patient‐specific bone tunnels (Table 1). This technique is based on the principles of “individualized bone tunnel planning, maximal bone preservation, and minimal physeal intervention” leveraging the advantages of the all‐inside approach. Preoperative MRI is used to accurately measure the ACL inclination angle and the distance from the anatomical footprint to the physeal line, enabling the design of a patient‐specific tunnel trajectory that facilitates anatomical ACL reconstruction with minimal physeal disruption. This technique has the following technical features: it first uses a small drill bit to penetrate the physis area on both sides to form a minimally invasive bone tunnel and then uses a larger drill bit to drill out a bone tunnel that does not penetrate the physis structure. This results in the physis intervention area being far less than 7% to 9% of the total physis area.^6^ Thus, this technique possesses several advantages (Table 2): it minimizes the damage area in the physis area to the greatest extent, reduces the risk of bone tunnel rupture during the operation, retains more bone volume, and achieves the anatomical reconstruction of the ACL. These advantages collectively ensure the normal growth potential of the physis structure and the restoration of knee joint stability. Furthermore, based on MRI‐derived measurements, appropriately sized bone tunnels are created at the ACL attachment sites. Adjustable suspensory fixation devices are used on both the femoral and tibial sides, with an additional knotless anchor employed on the tibial side for supplemental fixation. This configuration avoids graft or fixation hardware crossing the physis, thereby reducing physical interference, while providing robust fixation strength—promoting favorable tendon‐bone healing and biomechanical performance under conditions of minimal physeal intervention.

**TABLE 1:** Pearls and Pitfalls

Pearls	Pitfalls
High‐resolution MRI was used to accurately measure the distance between the ACL insertion point and the physis, as well as the ACL direction, in order to conduct patient‐specific bone tunnel planning.	Suboptimal MRI image quality or measurement inaccuracies may result in nonanatomical tunnel placement and length, thereby increasing the risk of iatrogenic physeal injury.
Use a smaller drill bit and try to pass through the centers of the physis perpendicularly, aiming for the smallest possible area of penetration.	Inaccurate control of the small drill's angle can lead to an enlarged transphyseal area or a poorly positioned bone tunnel for the tendon graft.
Adjustable suspensory fixation was employed on both the femoral and tibial sides to prevent the tendon graft and any interface screws from crossing the physeal area.	Improper placement of the suspensory device or suboptimal tension adjustment may lead to fixation failure or postoperative joint stiffness.
On the tibial side, supplemental fixation with a knotless anchor was performed to enhance initial stability.	If a large drill bit is used to create the bone tunnel for placing the tendon graft, and if the tunnel length is too long, it is very likely to pass through the physis area This results in more damage to the epiphyseal structures.

ACL, anterior cruciate ligament; MRI, magnetic resonance imaging.

**TABLE 2:** Advantages and Disadvantages

Advantages	Disadvantages
The design of patient‐specific bone tunnels ensures anatomical anterior cruciate ligament reconstruction while achieving the dual objectives of minimizing physeal intervention and maximizing bone preservation.	Although the bone tunnel traversing the physis is of small diameter, the technique is still classified as “transphyseal” and therefore carries a theoretical risk of physeal injury.
The creation of a minimally invasive tunnel traversing the central physis bilaterally in a near‐perpendicular orientation thereby lowers the risks of physeal shear damage and tunnel blowout.	This technique is restricted to patients aged 12 and above, with its applicability and safety in younger children not yet established.
It avoids the long‐term occupancy effect of the tendon graft and interference screw on the physeal area.	Long‐term, large‐sample follow‐up data are lacking to substantiate the long‐term outcomes and safety profile of the technique.

The present technique has several limitations that should be acknowledged. First, although the patient‐specific bone tunnel bilateral transphyseal all‐inside reconstruction minimizes physeal intervention, any procedure traversing the physis carries a theoretical risk of damaging the growth plate, potentially leading to limb length discrepancy or angular deformity. This risk becomes more pronounced if deviations occur in tunnel planning or execution. Second, the successful application of this technique heavily depends on the quality of preoperative MRI and the accuracy of measurements; suboptimal image quality or measurement errors may result in misplaced tunnels or incorrect tunnel lengths, thereby increasing the risk of physeal injury. Furthermore, there is currently a lack of large‐scale, long‐term follow‐up data to validate its long‐term functional outcomes and complication rates. Future prospective clinical studies are needed to further evaluate these aspects. Finally, this technique has thus far been applied only in adolescents older than 12 years with open physis at our institution, and its applicability and safety in younger pediatric populations remain to be assessed.

The patient‐specific tunnel technique presented in this study offers a viable solution for achieving anatomical ACL reconstruction, mitigating physical injury, and controlling graft‐related complications. The procedure is straightforward and associated with a short learning curve, making it readily adaptable and applicable for general orthopaedic surgeons.

## DISCLOSURES

The authors (Z.L., J.L, C.C., S.Z., H.F., H.W., J.Y.) declare that they have no known competing financial interests or personal relationships that could have appeared to influence the work reported in this article.
